# Rapid solar energy development in deserts: A missing element in desertification control and achieving Sustainable Development Goals

**DOI:** 10.1073/pnas.2601509123

**Published:** 2026-03-02

**Authors:** Hong Yang, Qi Feng, Jianhua Xiao, Guoshuai Li, Julian R. Thompson

**Affiliations:** ^a^State Key Laboratory of Ecological Safety and Sustainable Development in Arid Lands, Northwest Institute of Eco-Environment and Resources, Chinese Academy of Sciences, Lanzhou 730000, China; ^b^Department of Geography and Environmental Science, University of Reading, Reading RG6 6DR, United Kingdom; ^c^Department of Geography, University College London, London WC1E 6BT, United Kingdom

**Fig. 1.**
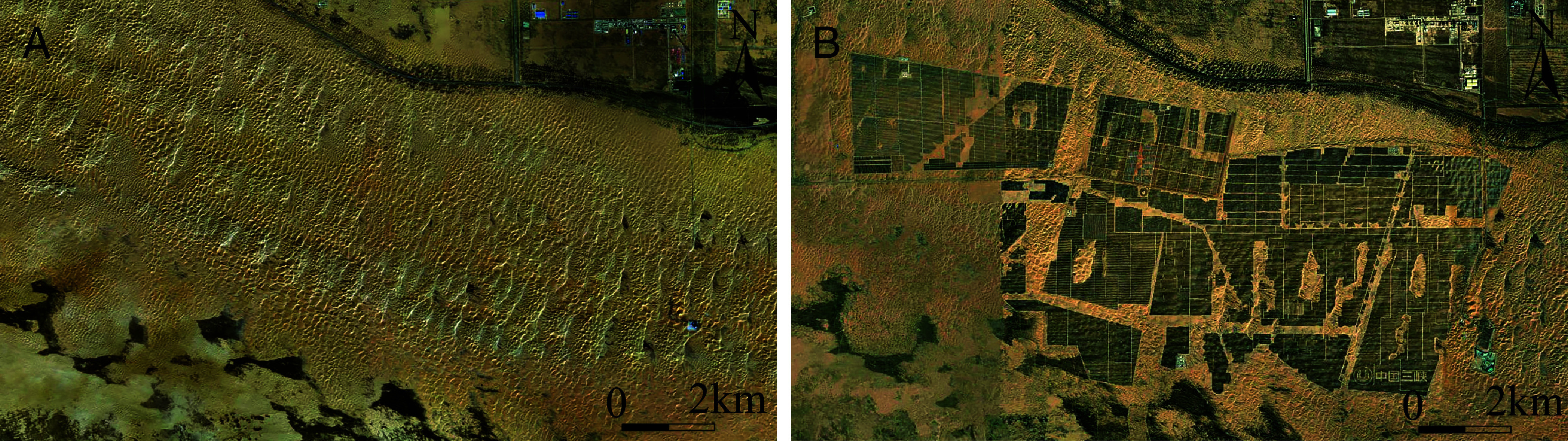
Rapid deployment of solar energy in desert regions illustrated by utility-scale photovoltaic installations in the Kubuqi Desert (Inner Mongolia, northern China), which expanded dramatically between 2018 (*A*) and 2025 (*B*). Remote-sensing imagery sourced from Esri World Imagery Wayback (https://livingatlas.arcgis.com).

Global desertification has attracted growing attention with Wang et al. recently highlighting land-use strategies for combating desertification and contributing to the Sustainable Development Goals (SDGs) 1, 2, and 6 (1). However, their analysis omits the rapid growth of large-scale solar energy development in deserts and its sweeping implications for the SDGs. With their low land costs and intense insolation, many deserts worldwide now host massive solar farms that bolster Affordable and Clean Energy (SDG 7). For example, utility-scale photovoltaic (PV) installations in China’s arid northern deserts expanded from nearly zero in 2011 to over 700 km^2^ in 2023 (2) (Fig. 1). This boom is not unique to China—similar large PV projects are underway across the Middle East, North Africa, and North America (3). Their potential benefits extend far beyond clean power.

No Poverty (SDG 1): Solar infrastructure in deserts can stimulate economic growth and improve local livelihoods. Desert-based PV programs in China have already improved residents’ welfare and spurred social prosperity in fragile sandy ecosystems (4). By creating jobs in construction and maintenance, solar farms provide new revenue streams in impoverished drylands. To fully realize SDG 1 gains, policymakers should integrate supportive measures including training, grid access, and revenue sharing for local communities—otherwise, poverty-reduction impacts of solar plants may be limited.

**Fig. 2.**
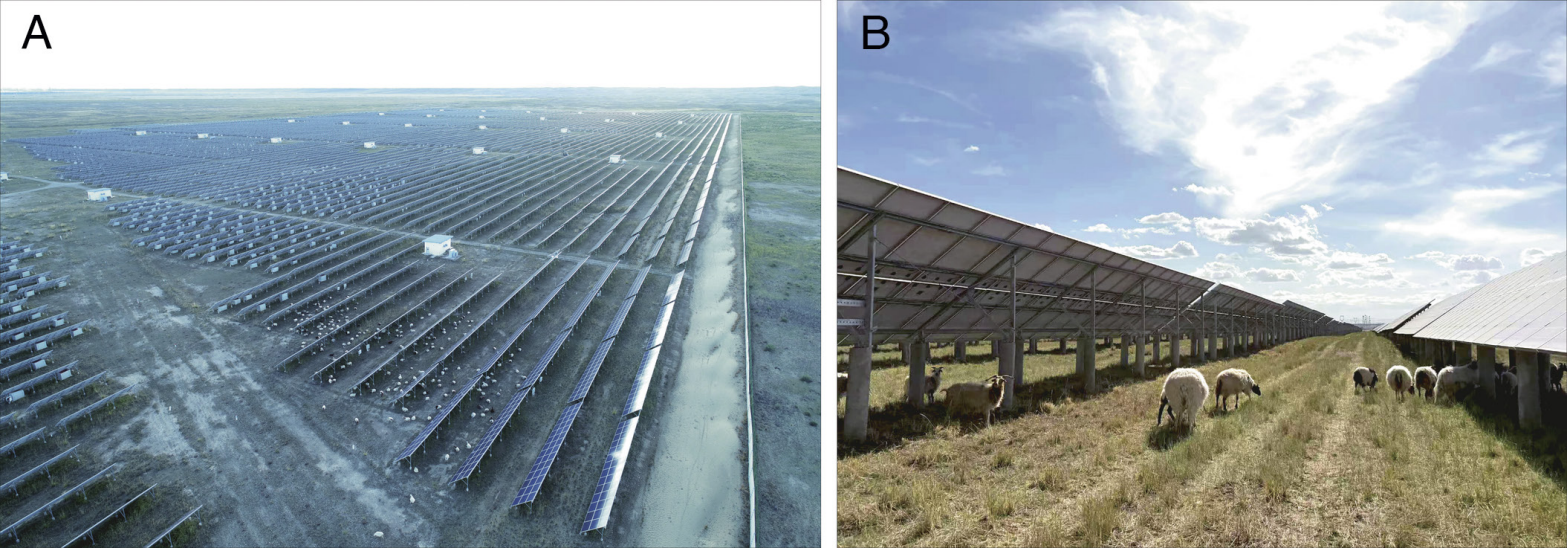
Solar farms can enhance soil moisture retention and stimulate grass growth (*A*), thereby improving forage availability and supporting livestock production (*B*), as demonstrated in Talatan Photovoltaic Park, Gonghe County, Hainan Tibetan Autonomous Prefecture, Qinghai Province, northwestern China.

Zero Hunger (SDG 2) and Clean Water (SDG 6): Far from competing with agriculture, desert solar can partner with it. Colocating PV with grassland and livestock (agrivoltaics) can provide reciprocal benefits in water-scarce environments. By retaining soil moisture, solar arrays can make desert farming more resilient and productive (Fig. 2). Solar-powered irrigation is another game changer: Solar pumps and smart drip systems can improve water-use efficiency by up to 70% and increase crop yields by 15 to 40%, directly contributing to SDGs 2 and 6 (5). Furthermore, solar-powered desalination can advance SDG 6 while simultaneously supporting SDGs 1, 2, and 7. This is exemplified by recent initiatives in the United Arab Emirates (6).

Fighting Desertification: Integrating solar energy into antidesertification programs can create positive feedback for land restoration. PV installations can physically aid land recovery—large solar farms in China’s deserts have increased vegetation cover (greening ~30% of project area) by shading soil and providing wind breaks (7). This “solar greening,” together with deliberate revegetation beneath and around photovoltaic arrays, can stabilize dunes and mitigate soil erosion. Modeling suggests that extensive PV installations in the Sahara could not only generate ample low-carbon electricity but also double rainfall and vegetation in the neighboring Sahel—potentially rejuvenating one of the most vulnerable dryland regions (8). However, very large desert arrays may also have other climate feedback (e.g., changing surface albedo and wind regimes) that warrant careful assessment and management (9).

Future research should explicitly consider large-scale solar development in deserts as an integral component of sustainable dryland management. Aligning renewable-energy deployment (SDG 13: Taking urgent action to combat climate change and its impacts) with established land-restoration initiatives could enable desert regions to function as engines of sustainable development rather than persistent zones of vulnerability (10).
